# A New Three-Hit Mouse Model of Neurodevelopmental Disorder with Cognitive Impairments and Persistent Sociability Deficits

**DOI:** 10.3390/brainsci14121281

**Published:** 2024-12-20

**Authors:** Imane Mouffok, Caroline Lahogue, Thomas Cailly, Thomas Freret, Valentine Bouet, Michel Boulouard

**Affiliations:** 1 Department of Health, Normandie Université, UNICAEN (Université de Caen Normandie), INSERM (Institut National de la Santé et de la Recherche Médicale), UMR (Unité Mixte de Recherche) 1075 COMETE, Campus 5, CYCERON, FHU (Fédération Hospitalo-Universitaire) A2M2P, CHU (Centre Hospitalo-Universitaire) Caen, 14000 Caen, France; imane.mouffok@etu.unicaen.fr (I.M.); caroline.lahogue@unicaen.fr (C.L.); thomas.freret@unicaen.fr (T.F.); michel.boulouard@unicaen.fr (M.B.); 2 CERMN UR (Unité de Recherche) 4258, Campus 5, Université de Caen Normandie, 14000 Caen, France; thomas.cailly@unicaen.fr; 3 CYCERON UAR (Unité d’Appui à la Recherche) 3408-US50, IMOGERE, Campus 1, Université de Caen Normandie, 14000 Caen, France; 4 Department of Nuclear Medicine, CHU Côte de Nacre, 14000 Caen, France

**Keywords:** psychosis, glutamate, phencyclidine, serine racemase, maternal separation, behavior, stress, social impairment

## Abstract

Background/Objectives: Cognitive deficits and negative symptoms associated with schizophrenia are poorly managed by current antipsychotics. In order to develop effective treatments, refining animal models of neurodevelopmental disorders is essential. Methods: To address their multifactorial etiology, we developed a new three-hit mouse model based on the hypoglutamatergic hypothesis of the pathology combined with early stress, offering strong construct validity. Thus, a genetic susceptibility (serine racemase deletion) was associated with an early environmental stress (24 h maternal separation at 9 days of age) and a further pharmacological treatment with phencyclidine (PCP, a glutamate receptor antagonist treatment, 10 mg/kg/day, from 8 to 10 weeks of age). The face validity of this model was assessed in female mice 1 and 6 weeks after the end of PCP treatment by a set of behavioral experiments investigating positive- and negative-like symptoms and cognitive deficits. Results: Our results showed that the three-hit mice displayed persistent hyperlocomotion (positive-like symptoms) and social behavior impairment deficits (negative-like symptoms) but non-persistent spatial working memory deficits (cognitive symptoms). Conclusions: Our work confirms the usefulness of a three-hit combination to model, particularly for negative-like symptoms associated with schizophrenia and other psychiatric disorders. The model therefore gathers powerful construct and face validities and supports an involvement of glutamate dysfunction in behavioral symptoms.

## 1. Introduction

Schizophrenia, affecting 1% of the global population, is a highly disabling psychiatric illness characterized by a wide range of symptoms classified into three types: positive (hallucinations, delusions, and disorganized thinking), negative (social withdrawal and emotional blunting), and cognitive (memory and attention deficits). The disease is associated with widespread morphological, neurochemical, and functional changes in the brain, associated with neuroplasticity deficits, in relation with its symptomatic features [1]. While positive symptoms are relatively well managed by current treatments, negative symptoms and cognitive deficits remain mostly unaffected by antipsychotic medication [2,3,4].

Rodent models are invaluable in the field of neuroscience research. However, animal models of schizophrenia are complex to design because of its unknown etiology, its multifactorial aspect, and the difficulty of mimicking high-level mental integration. Indeed, even if few multiple-hit models have been described (two-hit and three-hit), current animal models mainly focus on particular endophenotypes rather than mimicking the whole disease [5,6,7]. Whether positive symptoms are often investigated, studies are scarce regarding negative symptoms and cognitive deficits of the pathology.

Compelling evidence points to a deficiency in glutamate-mediated excitatory neurotransmission through N-methyl-D-aspartate (NMDA) glutamate receptors (NMDA-R) in schizophrenia [8,9,10,11]. The NMDA-R hypofunction hypothesis emerged from observations that subanesthetic doses of non-competitive NMDA-R antagonists, such as phencyclidine (PCP), ketamine, and dizocilpine (MK-801), induce psychotic reactions in healthy individuals and exacerbate symptoms in schizophrenic patients [9,12,13,14]. Consequently, NMDA-R blockade in rodents has been proposed as an animal model of the disease [15,16,17,18]. Indeed, non-competitive NMDA-R antagonists like PCP, ketamine, and dizocilpine have been shown to trigger schizophrenia-like positive and negative symptoms associated with cognitive deficits in rodents [17,19,20,21]. In particular, the model of subchronic administration of PCP in adulthood in mice is considered as a pertinent pharmacological model of schizophrenia with translational relevance [22,23,24,25]. Indeed, it has been shown in the literature that repeated administration of PCP is capable of inducing negative-like symptoms in rodents [17,26]. Regarding social behavior, numerous studies conducted in mice have demonstrated impairments [27,28,29,30], even if this is not always the case [31]. Furthermore, to our knowledge, the persistence of these negative-like symptoms is scarcely evaluated in animal models, while negative symptoms persist long term in patients [32].

Another important point is that even if the use of PCP induces some relevant behavioral effects, the PCP model does not fit with the hypothesis of a multifactorial etiology [33]. It therefore does not present an optimal construct validity (based on human pathogenesis) and provides limited value in explaining the etiopathology of schizophrenia. First described in 1977 by Zubin and Spring [33], the long-standing stress–vulnerability model suggests that schizophrenia arises from a combination of biological, social, and psychological factors. This gave an indication of a possible way to design animal models of this disease. A genetic predisposition, early stressor (such as maternal infection or stress), and later environmental influences (such as additional stress factors or substance use), can trigger schizophrenia-relevant endophenotypes [34,35,36]. In this context, we studied the face validity of a multifactorial three-hit model combining a subchronic PCP treatment in adulthood with a genetic susceptibility and a perinatal stress. More precisely, in order to get closer to the three-hit model and to reinforce the impact of NMDA-R hypofunction, we associated this pharmacological factor (subchronic administration of PCP in adult mice) as an environmental factor with a genetic predisposition (deletion of the serine racemase (sr) gene) and an early environmental stress (maternal separation).

The selection of the three hits was based on the following arguments. Several studies on the identification of genetic loci associated with schizophrenia have pointed toward several genes involved in glutamatergic neurotransmission [37,38,39]. Among these, *sr* has gained significant attention due to its critical involvement in SCZ pathology [37,40,41]. Serine racemase (SR) is an enzyme involved in the conversion of L-serine to D-serine, a co-agonist of NMDA-R. Clinical studies have uncovered polymorphisms in *sr* that are associated with reduced levels of D-serine in the cerebrospinal fluid and brain tissues of patients with schizophrenia [37,39,41,42,43]. Knock-out mice for this enzyme (SRKO) show a 90% reduction in cerebral D-serine concentration, leading to glutamatergic hypofunction associated with various disturbances found in schizophrenia [40,44,45,46,47]. We therefore used this *sr* deletion as a genetic factor predisposing to schizophrenia (first hit). A previous study reported that PCP-enhanced startle reactivity was increased in SRKO mice [48], suggesting a synergic effect of the association of this genetic deletion with a pharmacological treatment targeting NMDA-R.

Finally, we combined these two previously mentioned factors affecting glutamate neurotransmission with an early environmental stress: maternal separation (MS) from 24 h on the 9th day of postnatal life (second hit). Several studies have shown that early life stress, such as MS, profoundly impacts postnatal brain development, affecting the functional maturation of brain regions involved in cognitive and affective functions, which generates behavioral disorders relevant to mimicking certain symptoms of the pathology such as anxiety-like behavior and cognitive and sociability impairments [49,50,51].

We therefore aimed at characterizing this new three-hit model in female mice. Our choice to use female mice in this first single-sex study of a new three-hit model is based on several arguments. Psychoses gather several troubles with a sex prevalence that is approximately equal. However, most preclinical and clinical studies have been conducted in males; there is therefore a lack of data in females [52]. Moreover, some studies have found that female rodents exhibit higher behavioral sensitivity to PCP [53,54]. Finally, this choice is also in line with the 3Rs principle. Indeed, males from the same litters as the female mice used in this study have been allocated to another research project in the laboratory.

Our experimental design included behavioral assessment of positive-like, negative-like, and cognitive deficits. Furthermore, considering the fact that studies using NMDA receptor antagonists generally assess short-term behavioral deficits (approximately a maximum period of 3 weeks after pharmacological treatment), we carried out a protocol with two successive sessions to assess the robustness of the model, i.e., its capacity to induce long-term deficits. A first session was performed to assess the appearance of the deficits (session 1, starting one week after the last PCP administration) and a second session to assess the persistence of the deficits (session 2, starting 6 weeks after the last PCP administration).

## 2. Materials and Methods

### 2.1. Animals

This study involved wild-type C57BL/6J mice and SRKO mice originally issued from Coyle’s lab colony (Belmont, MA, USA) [40], sourced from the local animal facility (Centre Universitaire de Ressources Biologiques, University of Caen Normandy, Caen, France). The animals (*n* = 46) were housed in standard laboratory cages, with groups consisting of 4 to 5 individuals, under a reversed 12 h light/dark cycle (light on at 7:30 p.m.), and controlled temperature (21 ± 1 °C) and humidity (55 ± 10%). Food and water were provided ad libitum, and the environmental enrichment consisted of crinkle-cut shredded paper and a cardboard house. Animal identification was carried out post-weaning by placing a subcutaneous chip under anesthesia (isoflurane (5% for induction–2.5% for maintenance) in an O_2_/N_2_O mixture (0.3/0.7)). Behavioral assessments were conducted between 11 and 17 weeks of age, with weekly weight measurements. The experimental procedures conformed with the guidelines outlined by the French and European Economic Community for the ethical treatment and use of laboratory animals (2010/63/UE, project authorization n°49215-2024042615112665 v3). Every effort was made to minimize both the number of animals used and any potential suffering.

### 2.2. Maternal Separation (MS)

At the age of 9 days (postnatal day PND9), half of the wild-type and SRKO litters underwent a 24 h maternal separation [49,55]. Therefore, dams were removed and placed in individual cages near their pups. The following day, at 9:00 a.m., dams were placed back with their litters and left undisturbed until weaning. For the other half of litters (control mice), the separation period was only 20 s.

### 2.3. Pharmacological Treatment

Phencyclidine hydrochloride (PCP) (Sigma-Aldrich, Saint-Quentin-Fallavier, France) was dissolved in saline the day of the administration. The solution was subcutaneously (s.c.) administered at 8 weeks of age once a day at the dose of 10 mg/kg s.c, under a volume of 10 mL/kg for 14 days (method initially described by Noda) [56,57]. Control animals received saline injections for the corresponding period. Following PCP or vehicle treatment, animals underwent a one-week washout period before behavioral tests.

### 2.4. Behavioral Assessment

- General considerations

**Figure 1:**
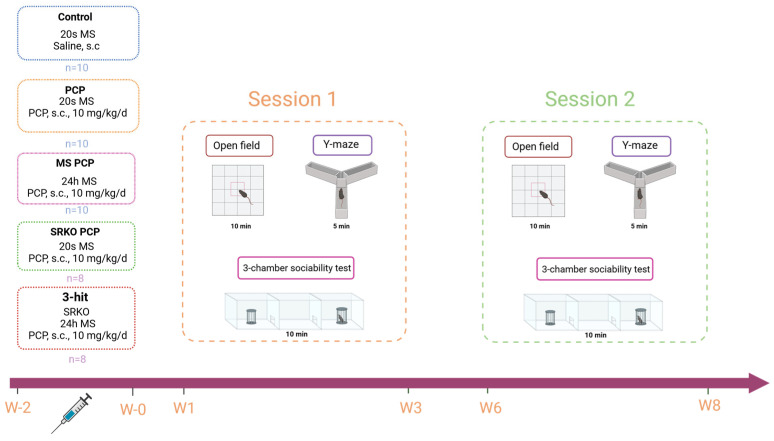
Animal groups and experimental design. Behavioral assessment was conducted 1 and 6 weeks after the end of PCP/saline treatment using a set of behavioral experiments investigating positive- and negative-like symptoms and cognitive deficits. PCP: phencyclidine; MS: maternal separation; SRKO: serine racemase knock-out; s.c: subcutaneous; W: week.

Animals were handled daily for 5 min during the week preceding the experiments. Behavioral tests were conducted from 9:00 a.m., corresponding to the beginning of the active dark phase of the cycle. Animal cages were placed in the experimental room 30 min before the beginning of each experiment. Apparatuses were cleaned with alcohol (70%) between each assessment. Two sessions of behavioral experiments, separated by 3 weeks, were performed (Figure 1). Two 2-week sessions of behavioral tests were conducted. The first started at 1 week after the end of PCP treatment at 17 weeks of age and the second occurred at 6 weeks after the end of PCP treatment at 11 weeks of age. The testing sequence of both sessions was identical, with the open field and Y-maze tasks in the first week and the 3-chamber sociability test in the second week. All behavioral tests were videotaped and scored using EthoVision XT v.16.0 (Noldus^®^, Wageningen, The Netherlands).

- Open field

Individual mice were placed in a white square arena (50 cm × 50 cm × 50 cm) for 10 min (15 lux) to assess spontaneous locomotion and exploratory and anxiety-like behavior. The total distance and percentage of time spent in the center of the open field (OF) were automatically collected [50,58].

- Y-maze

The Y-maze test was performed to assess spatial working memory according to previously established procedures [59,60]. Each mouse was placed for 5 min in the maze (20 lux), featuring three equally spaced arms (each sized 22 cm × 6.5 cm × 10 cm) and made of black-painted wood. Data collection included the number and order of arm entries (four-paw criterion). The alternation percentage was calculated using the following formula: number of alternations/(total number of entries − 2) × 100. Alternation percentages were compared to the reference value of 50%, representing random exploration of the maze and altered working memory.

- Three-chamber social interaction test

Social interaction was assessed using the three-chamber test, as previously documented [60,61]. The apparatus consisted of a Plexiglas box (60 cm × 45 cm × 22 cm) partitioned into three interconnected chambers (20 cm × 45 cm × 22 cm). Two cylindric wire cages (8.5 cm in diameter × 16.5 cm) were positioned in each side compartment, designated for placing another mouse and allowing visual and olfactory contact. Prior to testing, the subject mouse underwent a 5 min habituation period (20 lux), freely exploring all three chambers, with empty wire cages on both sides. Subsequently, an unfamiliar 4-week-old female mouse C57BL/6 mice (stimulus mouse), with no previous contact with the subject mouse, was placed in one of the side chambers within a wire cage allowing nose contact. The location of the stimulus mouse (left vs. right side chamber) was systematically alternated between trials. For 10 min, social behavior was investigated by collecting the cumulated time of interaction with the empty cage and the one with the stimulus mouse (direct contact or sniffing the cylinder and through the slits (nose less than 2 cm) and rearing up to the cylinder). The cumulated time of interaction with the wire cages was collected (direct contact or sniffing the cylinder and through the slits (nose less than 2 cm) and rearing up to the cylinder). Moreover, the distance traveled by the mice was also collected.

### 2.5. Statistical Analysis

Data were analyzed with GraphPad^®^ Prism 8.3. The normality of data distribution was assessed by Shapiro’s test and the homogeneity of variances by Levene’s test. For data normally distributed, group comparisons were performed using one- or two-way analysis of variance (ANOVA) followed by Dunett’s or Sidak’s multiple comparison test, respectively. For data not normally distributed, the Kruskal–Wallis test was performed. Results are presented as mean ± standard error of the mean (SEM) for normally distributed data and as median ± interquartile for not normally distributed data. Regarding spontaneous alternation data, a comparison with a threshold value of 50% was also carried out using a one-sample *t*-test. A significance threshold of *p* < 0.05 was considered for all analyses.

## 3. Results

### 3.1. Spontaneous Locomotor Activity and Anxiety-like Behavior

**Figure 2:**
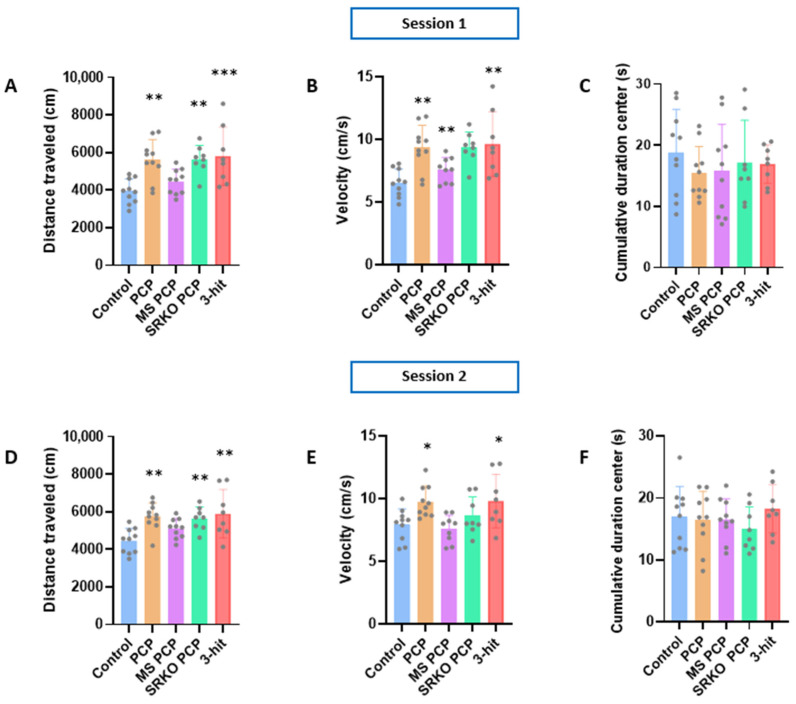
Spontaneous locomotor activity and anxiety-like behavior in the open field test during sessions 1 and 2 of the control (n = 10), 1-hit (PCP, n = 10), 2-hit (MS PCP, n = 10; SRKO PCP, n = 8), and 3-hit (SRKO MS PCP, n = 8) groups. Distance traveled (cm) in the open field arena during 10 min ((**A**,**D**) for sessions 1 and 2, respectively). Velocity (cm/s) in the open field arena ((**B**,**E**), for sessions 1 and 2, respectively). Total time (s) spent in the center of the open field ((**C**,**F**) for sessions 1 and 2, respectively). Data are presented as mean ± SEM. Grey dots represent individual data. Intergroup comparisons were performed using one-way ANOVA followed by Dunett’s multiple comparison test; * compared to control: * *p* < 0.05; ** *p* < 0.01; *** *p* < 0.001. PCP: phencyclidine; MS: maternal separation; SRKO: serine racemase knock-out; 3-hit: SRKO MS PCP.

During the first session, a significant group effect was observed for the distance traveled (one-way ANOVA *p* = 0.0002) and velocity (one-way ANOVA *p* = 0.0003). The distance traveled (Figure 2A) was significantly higher in the one-hit PCP (n = 10, *p* = 0.0014), two-hit SRKO PCP (n = 8, *p* = 0.0025), and three-hit groups (n = 8, *p* = 0.0009) than in the control group (n = 10). Furthermore, these three experimental groups showed a higher velocity (Figure 2B) compared to control group during the first session: one-hit PCP (*p* = 0.0017), two-hit SRKO PCP (*p* = 0.0022), and three-hit (*p* = 0.0016). During the second session, a significant group effect was observed for the distance traveled (one-way ANOVA *p* = 0.0013) and velocity (one-way ANOVA *p* = 0.0049). A significant increase in the distance traveled versus the control group (Figure 2D) was also observed for the three previously mentioned groups: one-hit PCP (*p* = 0.0022), two-hit SRKO PCP (*p* = 0.0099), and three-hit (*p* = 0.0013). Interestingly, only the one-hit PCP (*p* = 0.0273) and three-hit (*p* = 0.0341) groups presented a higher velocity compared to the control group during the second session (Figure 2E). Taken together, these results highlight a persistent alteration in spontaneous locomotor activity (distance traveled and velocity), underlining a hyperactive endophenotype in the one-hit PCP, two-hit SRKO PCP, and three-hit groups (Figure 2D,E). Interestingly, such a phenotype was not observed in the two-hit MS PCP group, neither during the first nor the second session, indicating that maternal separation prevented PCP-induced hyperlocomotion. Regarding the percentage of time spent in the center of the open field arena (Figure 2C,F for sessions 1 and 2, respectively), no significant difference was observed compared to controls for any group in both sessions. Therefore, none of the groups seemed to exhibit an anxious phenotype. This observation suggests that the alteration in spontaneous locomotor activity observed in certain groups seems not driven by alterations in anxiety-like behavior. 

### 3.2. Working Memory

**Figure 3:**
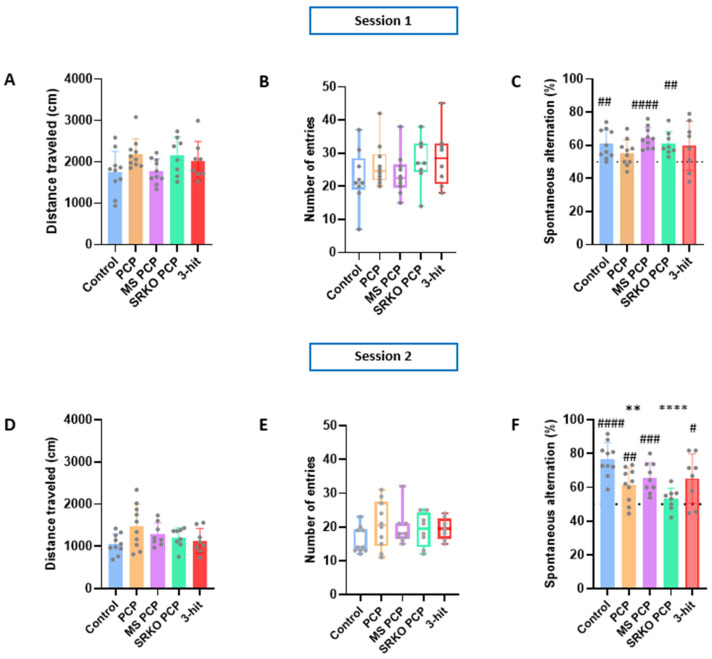
Spontaneous alternation in Y-maze test of controls (n = 10) and the 1-hit (PCP, n = 10), 2-hit (MS PCP n = 10; SRKO PCP, n = 8), and 3-hit (SRKO SM PCP, n = 8) groups. Distance traveled in the Y-maze for 5 min during session 1 (**A**) and 2 (**D**). Numbers of entries into the different arms during sessions 1 (**B**) and 2 (**E**). Spontaneous alternation percentage during sessions 1 (**C**) and 2 (**F**), with the dashed line represents 50% (i.e. chance level). Data are presented as mean ± SEM or as median ± interquartile. Grey dots represent individual data. Intergroup comparisons were performed using one-way ANOVA or the Kruskal–Wallis test followed by, respectively, Dunett’s or Dunn’s multiple comparison test; * compared to control: ** *p* < 0.01; **** *p* < 0.0001. # One-sample *t*-test Comparison to the reference value (50%) #: *p* < 0.05; ##: *p* < 0.01; ###: *p* < 0.001; #### *p* < 0.0001. PCP: phencyclidine; MS: maternal separation; SRKO: serine racemase knock-out; 3-hit: SRKO MS PCP.

Regarding spontaneous locomotor activity parameters, no significant difference in distance traveled during the Y-maze test was observed among the groups compared to the control for both the first and second sessions (Figure 3A,D). Similarly, there was no difference in the number of entries into the different arms of the apparatus for both the first and second sessions (Figure 3B,E). Regarding the first session, the ANOVA revealed no group effect in spontaneous alternation performances (Figure 3C). During the first session, the one sample *t*-test indicated that alternation percentages were not significantly above the 50% value for the one-hit PCP (n = 10, *p* = 0.0576) and three-hit (n = 8, *p* = 0.1069) groups, suggesting a spatial working memory impairment in these two experimental groups. However, these deficits were not observed during the second session (Figure 3F) regarding these two groups for which alternation percentages reached levels significantly above 50% (one sample *t*-test; one-hit PCP *p* = 0.0060; thre-hit *p* = 0.0217). Taken together, these results indicate a transient working memory impairment in the one-hit PCP and three-hit groups. Interestingly, the two-hit SRKO PCP group (n = 8), which displayed normal spontaneous alternation performances during the first session, displayed a spontaneous alternation percentage not significantly above 50% during the second session.

### 3.3. Social Behavior

**Figure 4:**
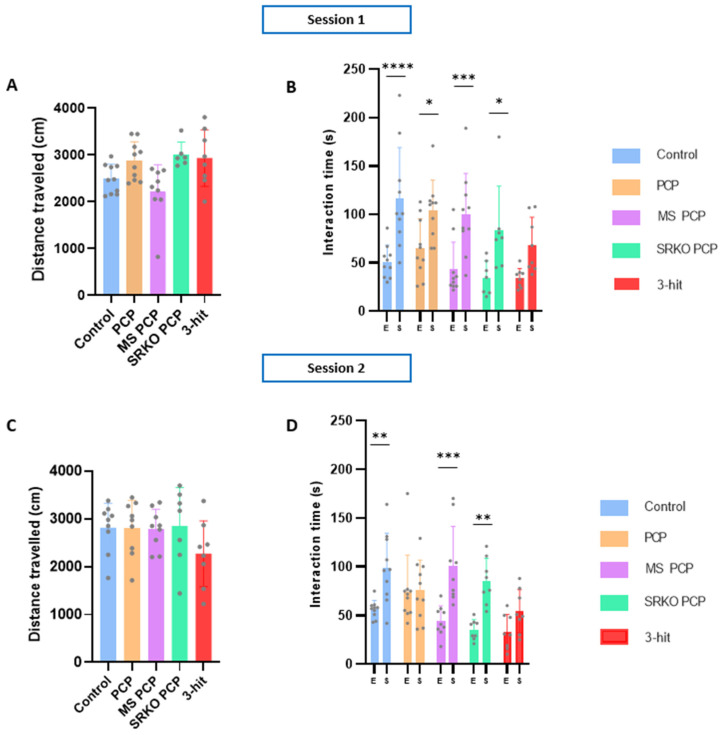
Social behavior of control (n = 10), 1-hit (PCP, n = 10), 2-hit (MS PCP, n = 10; SRKO PCP, n = 8), and 3-hit (SRKO SM PCP, n = 8) female mice. Distance traveled in the three-chamber apparatus for 10 min during session 1 (**A**) and 2 (**C**). Interaction time during sessions 1 (**B**) and 2 (**D**). Data are presented as mean ± SEM. Grey dots represent individual data. Intergroup comparisons were performed using two-way ANOVA with permutation followed by Sidak’s multiple comparison test. * E: empty vs. S: stimulus. * *p* < 0.05, ** *p* < 0.01, *** *p* < 0.001, and **** *p* < 0.0001. PCP: phencyclidine; MS: maternal separation; SRKO: serine racemase knock-out; 3-hit: SRKO MS PCP.

Regarding spontaneous locomotor activity parameters in the three-chamber apparatus, no significant difference in distance traveled was observed between groups for both the first and second session (Figure 4A,C). Concerning social behavior, a significant empty vs. stimulus effect was observed in interaction time during the first (two-way ANOVA *p* < 0.001) and second sessions (*p* < 0.001), but no significant group effect (Figure 4B,D). Except for the three-hit group, all other groups displayed social preference during the first session, attested by a significant difference in interaction time between the stimulus mouse and the empty cage (control (n = 10) *p* < 0.0001; one-hit PCP (n = 10) *p* = 0.0436; two-hit MS PCP (n = 10) *p* = 0.0006; two-hit SRKO PCP (n = 8) *p* = 0.0277; three-hit (n = 8) *p* = 0.2115). During the second session, the control, MS PCP, and SRKO PCP groups still displayed significant social preferences (control *p* = 0.0035; two-hit MS PCP *p* = 0.0001; and two-hit SRKO PCP *p* = 0.0020) but this was not the case for the three-hit (*p* = 0.4644) and one-hit PCP (*p* > 0.9999) groups. Indeed, as in the first session, the three-hit group showed a marked social deficit, characterized by a persistent lack of preference for the cage containing the mouse compared to the empty one (indicating impaired social behavior).

### 3.4. Summary of Experimental Data

**Figure 5:**
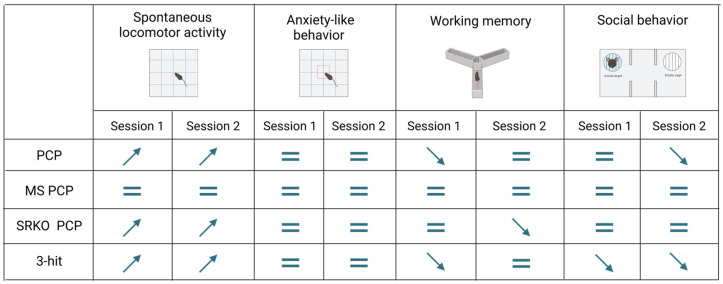
Behavioral assessment summary. Comparison of behavioral performances between each experimental group and control group. ↗ indicates a significant increase compared to control; ↘ indicates a significant reduction compared to control; and = indicates no significant difference compared to control. PCP: phencyclidine; MS: maternal separation; SRKO: serine racemase knock-out; 3-hit: SRKO MS PCP.

Figure 5 recapitulates the main behavioral results for each test and each session. Only comparisons to controls are depicted.

## 4. Discussion

The main objective of this study was to assess the face validity of models combining administration of subchronic PCP with another factor, either a genetic factor (deletion of *sr* gene) or a postnatal stress (maternal separation), or both, in female mice. The idea was to trigger in the initial pharmacological factor (PCP) more pronounced and persistent deficits, ultimately developing a robust three-hit model consistent with the multifactorial etiology hypothesis of schizophrenia. Our data indicated that the triple combination (three-hit) induced in mice a range of behaviors mirroring some hallmark schizophrenia symptoms. Indeed, the persistent hyperlocomotion observed in three-hit mice aligns with dopaminergic system dysregulation, often reported in patients experiencing psychosis, and can model positive-like symptoms of schizophrenia. Impairments in spatial working memory reflect the cognitive deficits frequently observed in schizophrenia. Additionally, the main finding of our study is the persistent impairment of social behavior, reflecting negative-type symptoms, known to drastically impact patients’ daily living. Our data moreover showed that adding a factor could in some case have protective effects, particularly concerning maternal separation.

The results will first be discussed regarding the short- and long-term effects of PCP administration in female mice. Then, the added value of a second factor (genetic predisposition or early stress) will be debated, before emphasizing the usefulness of the three-hit combination.

Very few studies have used subchronic administration of PCP in adult female mice [24,30,62,63]; for the most part, males are used. Unfortunately, differences in protocols used (in particular, PCP dose and/or duration of treatment, delay between treatment and behavioral assessment, or behavioral tests used) do not allow us to compare our results with those of these four studies. In our study, subchronic administration of PCP induced an increase in locomotor/exploratory activity in an open field 8 days after treatment cessation and this increase persisted for at least 6 weeks. In the same open field, there was no modification in anxiety-like behavior induced by PCP whatever the session considered. Furthermore, we observed that PCP induced a transient decrease in working memory performance in the spontaneous alternation test and a decrease in social behavior, which appeared only later, i.e., 7 weeks after the end of PCP treatment. Collectively, these data suggest the induction, by subchronic administration, of a behavioral phenotype characterized by robust positive-like symptoms (hyperlocomotion observed at the two sessions), associated with transient cognitive alterations (decreased working memory performance) and late negative-like symptoms (decreased sociability). Compared to the limited amount of literature data on the behavior of female mice after subchronic PCP administration, many studies have used male mice under these pharmacological conditions. Among these, some used a PCP exposure protocol close to ours (PCP dose, route of administration, number of injections, and duration of washout preceding behavioral tests), allowing us to compare our results, in particular concerning working memory performances and sociability behavior. In agreement with our data, Castañé et al. [64] reported a decrease in working memory performance (spontaneous alternation test) after 4 days of washout in male C57Bl/6J mice. Zhu et al. [65] also described impairment in spatial working memory even earlier, after one day of PCP treatment washout. Furthermore, Arime et al. [57,66] reported, with the same PCP treatment protocol and the same mouse strain as ours, a working memory deficit through another paradigm (the discrete paired-trial variable-delay task in a T-maze). These data and ours therefore suggest that the subchronic PCP administration protocol is suitable in female mice, as it is in male mice, to measure early working memory deficits (1 to 14 days after washout). They moreover indicate that this PCP-induced cognitive deficit is transient, no longer present 6 weeks after washout. The late effects on spatial working memory performances (beyond 6 weeks of washout) have, to our knowledge, not been studied to date.

Regarding sociability, several studies using strictly the same subchronic PCP administration protocol as us reported decreases in social behavior in male mice in the social interaction test [27,29,67,68,69]. Interestingly, Qiao et al. [64] showed that the deficit in social interaction was present as early as 3 days after the end of PCP administration and persisted at least until 28 days after the treatment, showing that both early and late deficits of social behavior are triggered by this PCP treatment. Such a result is only partially in agreement with our data (alteration in social behavior only in session 2). This difference could be related to the type of test used (three-chamber test versus social interaction test), the mouse strain (C57BL/6 versus ddY, ICR), or even the sex of mice. Our results suggest that a particular and progressive adaptation of the female mouse organism occurs in relation to the PCP regimen, underlying the delayed effect on social behavior we observed in session 2.

Understanding the interactions between different factors (synergy, resilience, and resistance) and the underlying mechanisms is essential [70]. Interactions can be classified into two major categories: synergy, in which a deficit induced by one factor is potentiated by another one, and resistance, in which a deficit induced by one factor prevents the deficit induced by another factor.

We focused firstly on the early-life stress factor, i.e., maternal separation, combined with subchronic PCP administration. Interestingly, adding this factor to PCP treatment restored a behavioral phenotype similar to that of the control group, counteracting the deleterious effects observed following chronic PCP administration. Some factors known to affect behavior do not always show expected schizophrenia-like symptoms when combined with other factors in two-hit and three-hit models. Indeed, maternal separation alone impaired social behavior but did not induce sociability deficits when combined with other factors [50,51,71]. Similarly, maternal separation alters sensory motor gating in rats [72], but this effect is not observed in combined models, highlighting the somehow protective effect of maternal separation [73]. Some studies have even shown that early stress, such as maternal separation, can be a protective factor, promoting resistance later in life [74]. To our knowledge, there is no study in mice that has associated maternal separation with chronic PCP treatment in adulthood. Interestingly, the study of Shin et al. [75] reported that another NMDA receptor antagonist, ketamine, when administered chronically to adolescent mice counteracted the anxiogenic and aggressive effects induced by neonatal maternal separation. This suggests that early maternal separation induces resistance to some behavioral alterations induced by a further non-competitive blockade of NMDA receptors. The results of our study seem to indicate that this resistance (MS-induced) could be extended to other types of behavioral alterations (spontaneous activity, working memory, and social behavior in particular). The molecular and cellular mechanisms underlying this resistance effect remain to be established.

Regarding the association of SRKO genetic susceptibility with repeated PCP administration, the resultant phenotype was relatively similar compared to the PCP model alone. Indeed, the addition of the genetic factor had no effect on the hyperlocomotion already obtained with one-hit PCP. The genetic deletion delays the memory deficit (deficit only during session 2) and prevents the alteration in social behavior induced by PCP treatment. This suggests that, to a lesser extent than neonatal maternal separation and genetic susceptibility, *sr* deletion would induce a phenomenon of resistance to the memory and social effects of chronic PCP administration. In that case, since the neurotransmitter system targeted by both the genetic deletion and the pharmacological treatment is the glutamatergic system, it can be argued that the genetic alteration would lead to an adaptation of this system, making it less sensitive to the effects of PCP. To our knowledge, no study to date has investigated the effects of chronic PCP administration in adult SRKO mice. Interestingly, Benneyworth et al. [48] showed that the behavioral effects (hyperactivity and blockade of prepulse inhibition of the acoustic startle reflex) of an acute dose of PCP were similar in WT and SRKO mice. This suggests that, under these pharmacological conditions (acute PCP administration), there is no synergistic effect between the two factors.

Regarding our three-hit model, the hyperactivity observed is not significantly different compared to one-hit PCP. Therefore, this observation suggests that there is no synergistic or additive effect following PCP administration when combined with *sr* deletion and MS. Interestingly, concerning cognitive deficit, the three-hit model displayed alterations in working memory performance (at the first session), which were not observed in both two-hit models. This suggests a complex interaction between the three risk factors, with the resistance of MS and *sr* deletion towards the PCP effect being reversed in the three-hit model. The main result of our study is the persistent sociability deficit in the three-hit model, suggesting that this model is particularly well suited to mimic the negative-like symptoms associated with various neurodevelopmental pathologies. Interestingly, a synergistic effect between the three factors was observed for this behavioral alteration.

An essential aspect of animal models, particularly for a chronic disease, is the robustness of deficits, i.e., the fact that deficits observed at one time persist over time. Moreover, given its chronicity, pharmacological treatments of schizophrenia are generally prescribed for a long-term duration, often for life, to stabilize the patient’s condition. Very few studies have characterized schizophrenia-like symptoms in preclinical models over several months like this study, filling the gap in the current literature. To assess the three-hit model’s robustness, we examined the cohort of female mice at two different ages: 2 and 4 months. We observed persistent hyperactivity in the three-hit mice, also observed in the one-hit PCP and two-hit SRKO PCP mice. This longitudinal behavioral assessment allowed us to identify more precisely the long-term effects of subchronic exposure to PCP. This type of pharmacological treatment effectively models positive-type symptoms in a durable and robust manner. Only the three-hit model showed a persistent sociability deficit across both behavioral assessment sessions. This is particularly interesting in view of the chronic and irreversible evolution of negative clinical symptoms [76].

The multifactorial etiology of schizophrenia [77] supports the tuning and validation of new three-hit animal models with powerful construct validity. The behavioral alterations reported in three-hit studies [50,51,70,71] suggest that this combined strategy is particularly appropriate for modeling schizophrenia etiology, particularly for negative symptoms and cognitive deficits, which are currently unsupported. Understanding the interactions between different factors and the underlying mechanisms will enable development and assessment of new pharmacological strategies.

While our findings reinforce the construct and face validities of the three-hit model in replicating schizophrenia-like phenotypes, several limitations may be noted. The first one is related to the sample size. Indeed, a larger n could have strengthened the effects observed and maybe highlighted others. However, it aligns with similar published studies regarding other schizophrenia models [78,79,80]. Indeed, the complexity of the model construction and the 3R principle compelled us to use a low number of animals but, in spite of this, statistical analyses allowed us to extract significant effects. Another limitation is related to the absence of subjective reporting in animals, which restricts our ability to confirm whether altered behaviors accurately parallel clinical observations such as hallucinations or delusions, central to schizophrenia. Additionally, findings from pre-clinical studies can sometimes be difficult to translate into clinical settings due to species-specific differences in neurobiology and behavior. Furthermore, while robust in displaying social behavior impairment, a broader spectrum of negative-like symptoms could be investigated in the three-hit model, by testing anhedonia and avolition.

In conclusion, our findings bolster the construct and face validities of the three-hit model, replicating core schizophrenia-like phenotypes across positive, cognitive, and particularly robust negative symptom domains. This comprehensive symptom profile makes the model valuable for further investigation of the complex neurobiological underpinnings of schizophrenia, and of new therapeutic strategies, especially in the treatment of negative symptoms. Additionally, the model implicates glutamate dysregulation in the manifestation of behavioral symptoms, mirroring the current understanding of schizophrenia pathophysiology [81,82]. Further research exploring other negative-like symptoms and the predictive validity of the model will be crucial for its continued development and refinement.

## Data Availability

The data that support the findings of this study are available from the corresponding author upon reasonable request.
